# Characterization of the Lithium/Solid Electrolyte Interface in the Presence of Nanometer‐thin TiO_x_ Layers for All‐Solid‐State Batteries

**DOI:** 10.1002/cssc.202401026

**Published:** 2024-10-16

**Authors:** Rainer Götz, Ekaterina Pugacheva, Zahra Ahaliabadeh, Princess Stephanie Llanos, Tanja Kallio, Aliaksandr S. Bandarenka

**Affiliations:** ^1^ Physics of Energy Conversion and Storage Physics Department Technical University of Munich James-Franck-Str. 1 85748 Garching Germany; ^2^ Electrochemical Energy Conversion Aalto University P.O. Box 11000, Otakaari 1B Aalto 00076 Finland

**Keywords:** All-solid-state batteries, Interlayer, Lithium metal anode, Solid-solid interface, Space charge layer

## Abstract

It is still unclear which role space charge layers (SCLs) play within an all‐solid‐state battery during operation with high current densities, as well as to which extent they form. Herein, we use a solid electrolyte with a known SCL formation and investigate it in a symmetric cell under non‐blocking conditions with Li metal electrodes. Since the used LICGC™ electrolyte is known for its instability against lithium, it is protected from rapid degradation by nanometer‐thin layers of TiO_x_ deployed by atomic layer deposition. Close attention is given to the interfacial properties, as now additional Li^+^ can traverse through the interface depending on the applied bias potential. The interlayer‘s impedance response shows efficient lithium‐ion conduction for low bias potentials and a diffusion‐limiting effect towards high positive and negative potentials. SCLs grow up to a thickness of 5.1 μm. Additionally, estimating the apparent rate constant of the charge transfer across the interface indicates that the potentials where kinetics are hindered coincide with the widest SCLs. In conclusion, the investigation under higher steady‐state currents was only possible because of the improved stability due to the interlayer. No chemo‐physical failure could be observed after 800+ hours of cycling. However, an ex‐situ SEM study shows a new phase at the interface, which grows into the electrolyte.

## Graphical abstract

The study employs nanometer‐thin TiO_2_ as an interlayer (IL) between lithium metal and the solid electrolyte LICGC. This allows the electrochemical characterization of the thereby stabilized interface. Besides limiting the kinetics towards high applied potentials, the interlayer also leads to broad space charge layers.

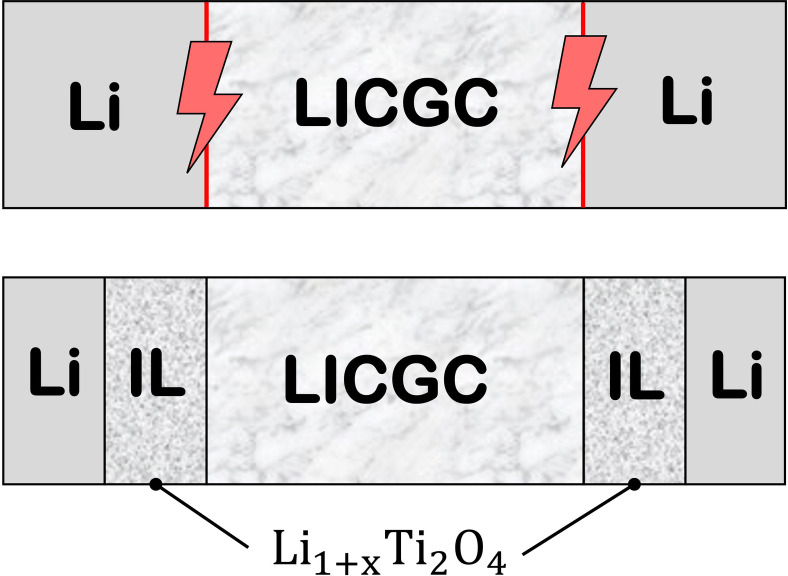

## 1 Introduction

All‐solid‐state batteries (ASSBs) could become a notable advancement in energy storage technology, promising higher energy densities, enhanced safety, and longer lifespans compared to traditional lithium‐ion batteries.^[1]^ As ASSBs approach the verge of commercialization, critical challenges remain that hinder their widespread adoption. Notably, the interface between the electrodes and the electrolytes presents a multi‐faceted obstacle as interface impedance can arise due to improper contact and instability at the interface, causing dendrite formation.^[2]^ An additional phenomenon at the electrode‐electrolyte interface is the formation of space charge layers (SCLs). For an electrolyte under the so‐called blocking conditions (no charge transfer across the electrolyte interface due to a blocking metal layer like gold), SCLs can extend hundreds of nanometers into the electrolyte.^[3]^ A thorough understanding of the role and the thickness of SCLs within a complete battery setup, which means moving to non‐blocking conditions, is essential to comprehend their influence on ion transport and overall battery operation, thereby enabling the optimization of ASSB designs for efficient and stable energy storage systems.

Most oxide solid electrolytes and the unprotected Ohara model electrolyte (LICGC™) used in this study are usually not very suitable for long‐term operation in ASSBs due to their instability against Li metal.^[4]^ Consequently, direct contact leads to increasing interface impedance, the formation of cracks,^[4]^ and eventually shattering of the electrolyte within hours (at ~2 bar cell pressure), as our preliminary tests for this study have shown. As such, it behaves similarly to other NASICON‐like lithium‐ion conducting electrolytes.^[4]^ Different approaches are known to mitigate interface issues and enhance battery stability at solid‐solid interfaces. Two notable strategies include the use of composite electrolytes^[5]^ and interlayers composed of metals or metal oxides.^[6]^ From the latter, we focus on titanium dioxide (TiO_2_). Studies suggest that amorphous TiO_2_ undergoes the following reaction during Li^+^ intercalation:[^7^, ^8^]

(1) $$\mathrm{Ti}O_{2}+\mathrm{xL}i^{+}+xe^{-}\to Li_{x}\mathrm{Ti}O_{2}$$

As shown previously[^9^, ^10^, ^11^, ^12^] the general composition of Li_x_TiO_2_ can be divided into individual phases, depending on the lithiation degree and, hence, the fraction of lithium *x* within Li_x_TiO_2_. Kuhn et al. point out that for most of the compositions, ramsdellite‐type Li_x_Ti_2_O_4_ consists of two mixed phases and that there are only a few small regions where the structure displays a single phase.^[12]^ For example, the compositional fraction of the third phase appears at x = 1.32 and continues to rise thereafter. However, it is only within a very narrow range (1.9 < x ≤ 2.0) near the end member Li_2_Ti_2_O_4_, that the material is exclusively composed of the third phase. As a close investigation from Gao et al. proves, though, DFT calculations favor Li_2_Ti_2_O_4_ since the Gibbs free energy reaches a minimum for x = 2. Additionally, after the complete lithiation, the amorphous Li_2_Ti_2_O_4_ can spontaneously crystallize into cubic Li_2_Ti_2_O_4_.^[8]^ Finally, all studies show excellent reversibility and low volume change upon de‐/intercalation for amorphous Li_x_TiO_2_ (~4 %) and especially for (cubic) Li_2_Ti_2_O_4_ (~0.9 %), which is a desirable characteristic for electrodes as well as protection layers thereby keeping the physical integrity of the interface upon repeated charging and discharging cycles.

As liquid electrolytes are also known for their instability against metallic lithium, Wang et al. discovered that an ultrathin layer of TiO_2_ on metallic lithium forms a stabilizing layer in a symmetric Li/TiO_2_/liquid electrolyte/TiO_2_/Li cell configuration. As described above (**Equation 1**), they suggest that lithium metal in contact with the TiO_2_ layer transforms the latter into cubic Li_2_Ti_2_O_4_, an efficient Li^+^‐ion conductor. Hence, dendritic growth is suppressed, leading to a notable enhancement in the lifespan of the symmetric cell when the TiO_2_ layer thickness is maintained within an optimal range, ideally around 5 nm, avoiding both excessive thickness and thinness.^[13]^

This study evaluates comparable TiO_2_ layers as interlayers between a Li metal anode and a solid Li‐ion conducting Ohara‐glass (LICGC) electrolyte for ASSBs. By stabilizing this interface, the cell can be analyzed by Staircase Potentiostatic Electrochemical Impedance Spectroscopy (SPEIS). This rather lengthy method applies a bias potential for a given time, then records an impedance spectrum, and moves on to the next potential. The quality of the data is heavily influenced by the stability of the cell within both the potentiostatic waiting time as well as during the impedance spectroscopy. Hence, to get reliable impedance data, the cell should be as stable as possible to avoid dynamic influences from, e. g., degradation processes of the solid electrolyte and its interface, which impair the analysis of SCL formation and, in general, the interfacial properties.

Compared to previous studies on the SCL effect within LICGC, we want to focus here on measurements under non‐blocking conditions with higher steady‐state currents, enabled by stabilizing TiO_2_ interlayers. Specifically, close attention is given to the corresponding influence of both the higher currents and the modified interface on the SCL formation.

## 2 Results and Discussion

### 2.1 Lithiation Phase

**Figure 1:**
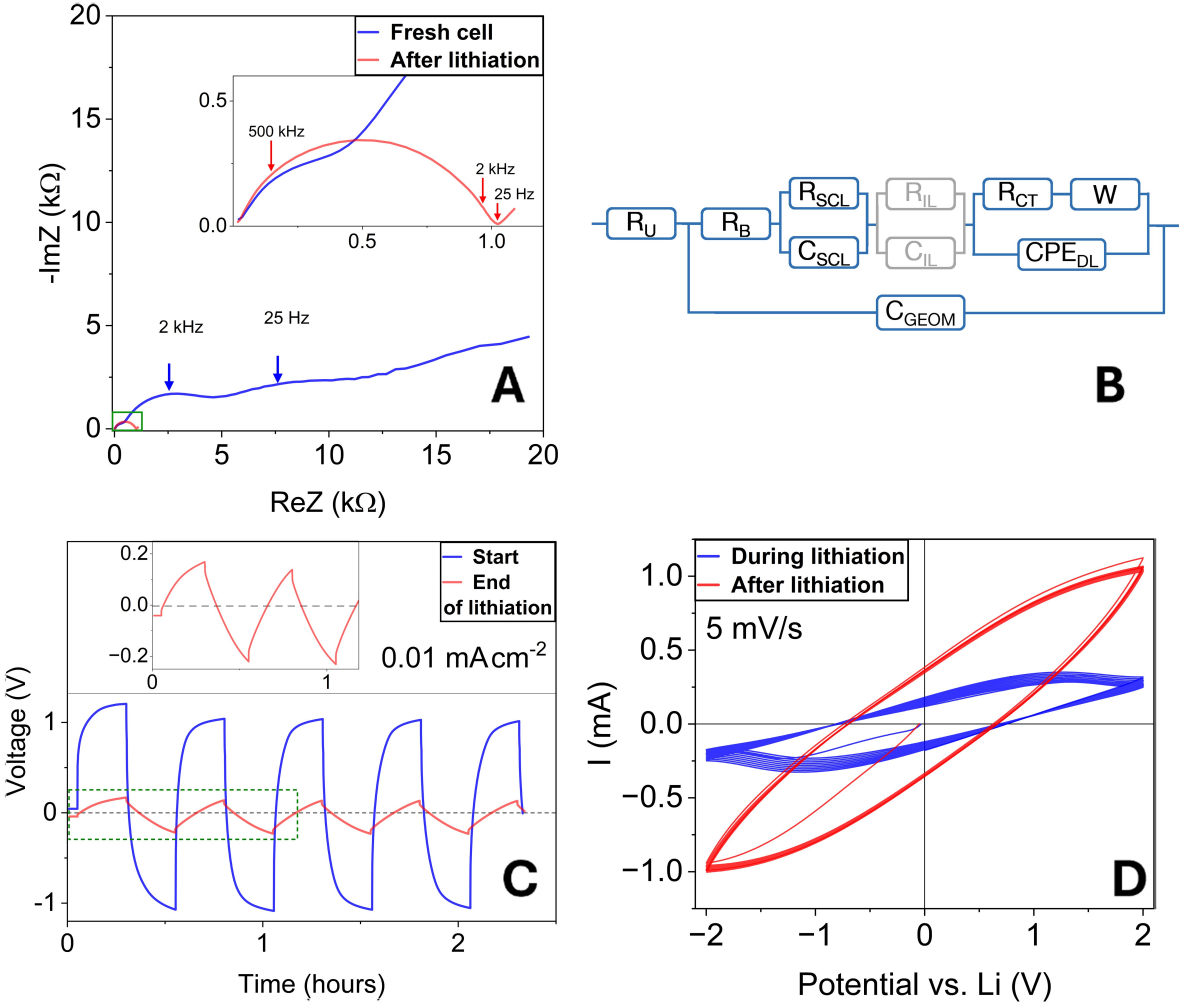
(A) Impedance data of the 5 nm sample at 0 V before (blue) and after lithiation (red). Green rectangle indicates the region of the zoomed inset picture (B) Electrical equivalent circuit (EEC) for a Li/TiO_2_/SSE/TiO_2_/Li cell configuration (see text for explanations) (C) Galvanostatic constant current cycling with a current density of 0.01 mA cm^−2^ of the cell with a 5 nm TiO_2_ layer (5 nm sample). Comparison between the start of the lithiation (blue) and at the end of it (red). Inset shows a zoom of the green dotted rectangle. (D) Cyclic voltammograms between −2 V and+2 V during (blue) and at the end of the lithiation phase (red) for the 5 nm sample.

The Nyquist plot from the initial EIS measurement at 0 V of the freshly assembled 5 nm cell shows a semicircle at high frequencies followed by larger semicircles in the mid and low‐frequency range (Figure 1A). The latter can be attributed to the initial low conductivity of the TiO_2_ interlayer and interfacial impedances stemming from improper contact between the lithium discs and the coated electrolyte, all of which are expected to decrease after the first lithiation cycles.[^6^, ^14^]

From previous studies, the standard glass‐ceramic electrolyte LICGC samples show a bulk resistance between 60–100 Ω[^15^, ^16^] for Au‐contacted samples under blocking conditions, and around 140 Ω in a non‐blocking cell setup.^[3]^ Similarly, after assembly, the analysis using the equivalent circuit (Figure 1B, which is explained later) yields approximately 175 Ω for the bulk resistance at 0 V, which roughly remains the same after lithiation. At the same time, the mid‐ and high‐frequency impedance contributions shrink (cf. Figure 1A) and the potential during galvanostatic charge‐discharge (CC: 10 μA/cm^2^) decreases from 1.2 V initially to about 0.2 V after cycling (cf. Figure 1C). Both observations match with the lithiation of the TiO_2_ interlayer, as it is supposed to transform into a lithium titanate with a higher lithium‐ion conductivity compared to the original material^[13]^ (cf. Table 1).

**Table 1:** *Diffusion coefficients of TiO_2_ and Li*_*1+x*_ *Ti_2_O_4_ depending on the lithium content x*.

Material	Diffusion coefficient	Reference
TiO_2_ (at 1.5 V)	1.5 – 6.0 ⋅ 10^−12^ cm^2^/s	[17]
Li_1+x_Ti_2_O_4_ (x = 0…1)	0.5 – 3.6 ⋅ 10^−11^ cm^2^/s	[18,19]

Cyclic voltammograms during and after the lithiation phase indicate the ongoing Li^+^‐intercalation (cf. Figure 1D). The slightly lower intercalation potentials compared to bulk TiO_2_ are characteristic of thin films^[17]^ and additionally point towards a constant potential throughout the TiO_2_ layer.^[31]^ After the lithiation, the maximum current increased from around 0.25 mA to approx. 1 mA at ±2 V (cf. Figure 1D). Based on these observations, we conclude that the lithiation is complete. To emphasize this, the layer between the Li metal and the solid‐state electrolyte (LICGC) will be called *interlayer* (IL) from now on.

### 2.2 Characterization Phase

To characterize the as‐prepared samples, SPEIS was used to differentiate between the individual elements within the electrical equivalent circuit (cf. Figure 1B) at varying potentials vs. Li metal.

**Figure 2:**
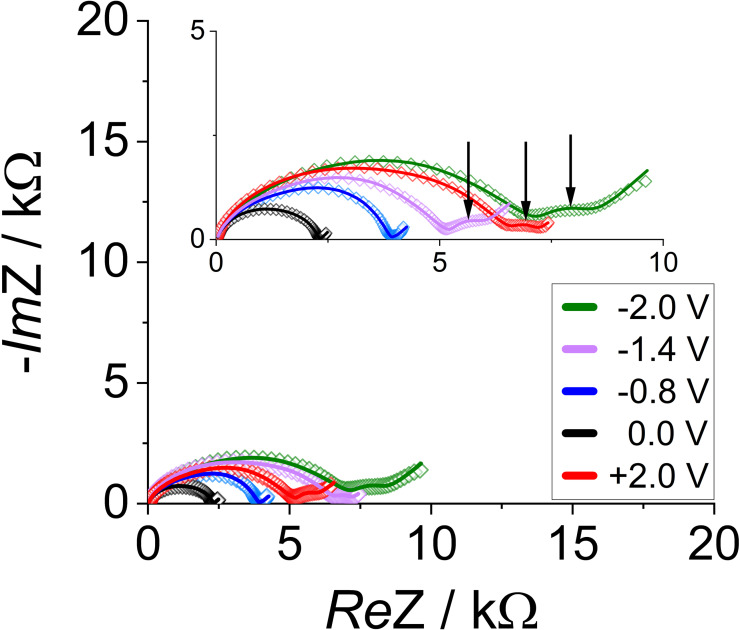
Fitted Nyquist plots of the cell with a 5 nm TiO_2_ layer after lithiation and for selected potentials vs. Li. The non‐negligible response of the interlayer leads to an additional semicircle (inset: arrows), which appears from −2 V to −0.8 V and then again from +1.6 V to +2.0 V. Representatively shown here are plots for −2.0 V, −1.4 V, +2.0 V as examples for spectra with a pronounced additional semicircle and 0.0 V without one. A vanishing semicircle is hardly visible for −0.8 V.

In a first overview, one can see that at frequencies around 4 kHz, another semicircle emerges when high bias potentials are applied (cf. Figure 2). This motivates the use of a more comprehensive equivalent circuit (cf. Figure 1B), including uncompensated resistances from the cell setup (R_U_), resistances attributed to the bulk (R_B_), the depletion space charge layer (R_SCL_), and charge transfer (R_ct_) and their respective capacitances: geometric (C_GEOM_), depletion space charge layer (C_SCL_) and double layer capacitance (C_DL_).[^3^, ^15^] An additional Warburg element in series with R_ct_ describes the diffusion limitation, especially towards the mentioned higher bias potentials.

Moreover, the interlayer gives rise to another RC‐combination: R_IL_ and C_IL_. Since the response from the lithiated interlayer becomes negligible between −0.8 V and +1.6 V (cf. Figure 2), the R_IL_C_IL_‐part is omitted within this potential window (cf. Figure 1B, grey elements). As one can see from Figure 2, the model gives an appropriate fitting. We will show that the interlayer can be considered highly ionically conductive within the range where no additional semicircle is visible. At the same time, diffusion limits the Li^+^ conduction through the interlayer for higher bias potentials. However, as the used model either includes the additional RC‐circuit or it does not, the fit is not optimal at the transition to an additional semicircle, as the fitting cannot incorporate the vanishing semi‐circle (cf. Figure 2, −0.8 V). As the parameters of the interlayer will show, this leads to high errors at potentials close to transition.

**Figure 3:**
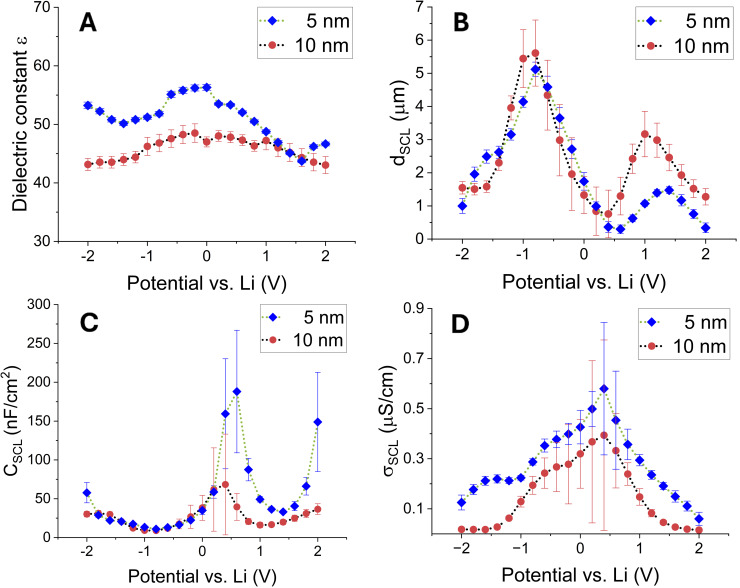
(A) Total dielectric constant ε of the cells derived from C_GEOM_ (cf. Figure S1B), hollow circles represent data points with high errors. (B) Space charge layer thickness d_SCL_ calculated from the charge layer capacitance C_SCL_ (C). And (D) the SCL′s ionic conductivity σ_SCL_ (derived from R_SCL_, cf. Figure S1C).

Compared to a solid electrolyte with blocking gold contacts, where the bulk properties of the solid electrolyte (R_B_ and C_GEOM_) remain constant with varying applied bias potential,[^15^, ^16^] the non‐blocking cells investigated here show changes in both elements (cf. Figure 3A and S1A) within the potential range under investigation (from −2 V to +2 V). Compared with a commercial LICGC sample under blocking conditions with an ionic conductivity σ_bulk_ of 1.0 ⋅ 10^−4^ S/cm at room temperature, the σ_bulk_ obtained in this study ranges from 0.3 ⋅ 10^−4^ S/cm (at −0.4 V) up to 1.7 ⋅ 10^−4^ S/cm (at +1.6 V). Hence, the applied potential either depletes or enriches the solid‐state electrolyte with Li^+^. The same can be seen in a non‐blocking cell with an identical electrolyte where a gold‐lithium‐electrode injects Li^+^ ions into the electrolyte.^[3]^

The dielectric constant *ε* of the constructed cells can be approximated with the geometric capacitance (cf. Figure S1B) by using *ε* = *dC_GEOM_* /*ε_0_A*, where *d* is the combined thickness of interlayer and solid electrolyte (≈150 μm), *ε_0_* the vacuum permittivity, and *A* is the contact area of the metallic lithium discs (1.7 cm^2^). This yields values that are about ten times smaller (*ε* = 44 – 56*)* compared to an Au/LICGC/Au cell under blocking conditions (*ε* = 677*)*.^[15]^

As can be seen in Figure S2C, the double layer capacitance (CPE_DL_) symmetrically increases with the potential, indicating a thinning of the double layer (DL), and then reaches a plateau towards both high negative and high positive bias potentials, resembling a Stern‐Helmholtz‐like capacitance behavior. It correlates with results found in previous studies but shows a lower capacitance compared to an Au/LICGC interface.[^16^, ^20^] Also, the *R_ct_* and Warburg impedance *W* heavily increase for applied bias potentials beyond ±1 V (cf. Figure S2A–B). As depicted in Figure S1C, the resistance of the SCL (depletion layer), *R_SCL_*, grows for higher applied bias potentials and then either plateaus (10 nm sample) or even decreases (5 nm sample). In contrast, the SCL capacitance (depletion layer), *C_SCL_*, has the opposite behavior (cf. Figure 3C). From *C*_*SCL*,_ one can approximate the depletion SCL thickness using *d_SCL_* = *ε_0_εA/C_SCL_*, assuming the dielectric constant remains the same throughout the sample. Although this way of determining SCL properties proved to be useful under blocking conditions,[^15^, ^16^] applying such a method to non‐blocking samples comes with a flaw. According to the latest study by Katzenmeier et al.,^[3]^ the dielectric constant changes with increasing/decreasing lithium content within the solid electrolyte. By using the permittivity of the original bulk (*ε* = 677) for the approximation of d_SCL_, one ignores this influence of the varying Li^+^‐content and, hence, the changing dielectric constant. This leads to an overestimation of the space charge depletion layer thickness, determined by means of EIS. The corresponding study obtains SCL thicknesses up to 600 nm in a potential range of ±1 V by EIS but only up to 400 nm via spectroscopic ellipsometry.^[3]^

Here, the sample exhibits lower dielectric constants after the lithiation phase, which is supposedly due to the lithiation of the TiO_2_ layer, as the dielectric constant decreased over time during the lithiation phase. The changes in permittivity when different potentials are applied can be attributed to varying lithium concentrations within the solid electrolyte. Meanwhile, the bulk resistance follows accordingly (cf. Figure 3A and S1A). Consequently, using the original relative permittivity of the bulk electrolyte is no longer a valid assumption for the studied samples.

Hence, for the approximation of the depletion SCL thickness, we herein utilize the *in‐situ* determined dielectric constant (ε = 44 – 56) at its respective potential (cf. Figure 3A). This leads to SCL thicknesses ranging from 0.30 μm to 5.1 μm between −2 V and +2 V (cf. Figure 3B). An asymmetry in the height between the two SCL peaks at −0.8 V (ca. 5.1 μm) and +1.4 V (ca. 1.5 μm) is also noticeable. Although such a finding is common in experiments, where it is explained due to the mentioned overestimation based on a varying dielectric constant Li^+^‐ concentration,^[3]^ it is also a consequence of the experimental setup: If, as it is the case for the spectroscopic ellipsometry study by Katzenmeier et al., only one sample surface can be examined, then the applied potential creates, respectively, an accumulation layer for a negative bias voltage or a depletion layer for positive bias voltage at the examined side. The accumulating positively charged lithium ions repel each other, leading to a broader depletion SCL compared to the accumulation side.^[16]^ Depending on the applied bias, one either observes the wider accumulation layer or the thinner depletion layer.

**Figure 4:**
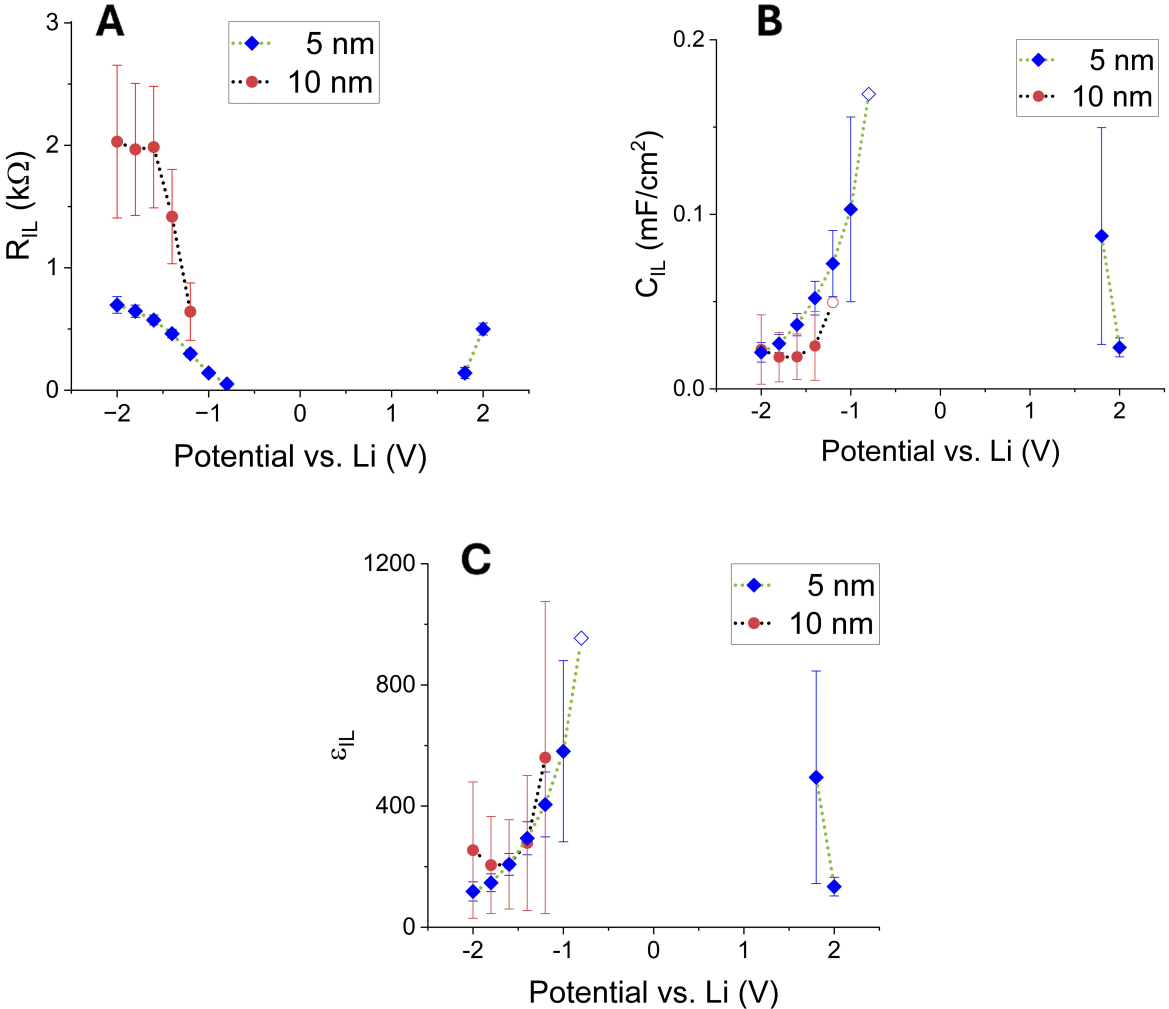
Interlayer parameters plotted versus the applied bias potential. (A) Resistance R_IL,_ (B) capacitance C_IL_ and (C) dielectric constant of the interlayer ε_IL_. Hollow data points have high errors due to the transition zone between the two used models (see description in the text).

Electrochemical impedance spectroscopy, however, can only probe the dielectric nature of the depletion layer; hence, a symmetric SCL profile with respect to the applied bias potential would be expected. The asymmetry in SCL width, along with the shifted symmetry axis (around +0.5 V instead of 0.0 V), as well as the asymmetric behavior of *R_ct_* and *W* (see Figure S2A and S2B), suggest a slightly asymmetric sample structure. However, the comparison between the samples shows that the SCL thickness is in the same range (cf. Figure 3B). The same goes for parameters like *R_Bulk_* or *C_GEOM_* hinting that the thickness of the interlayer itself does neither influence bulk properties nor the SCL width. On the other hand, properties that describe the Li^+^ movement across the interface, like *R_ct_* and the Warburg element *W*, show the increasing diffusion limitation towards higher potentials for a thicker interlayer (cf. Figure S2A–B). Also, the interlayer resistance *R_IL_* is always higher for the 10 nm interlayer (cf. Figure 4A).

Moreover, the interlayer/LICGC interface modifies the SCL compared to an Au/LICGC interface like in Katzenmeier et al.′s setup. The latter can observe space charge layers that are thinner (up to 600 nm) and have a much higher resistance (up to 200 kΩ) than the observed SCLs in the 5 nm sample (up to 5.1 μm thick and up to 1 kΩ resistance). Since the maximum SCL thickness is accordingly more than 8x bigger, the vacancy concentration on the depletion side (SCL) and, correspondingly, also the Li^+^‐concentration on the accumulation side have to be less dense to explain the low SCL resistance in this case. This is in line with the presumption that both an SCL close to depletion and, at the same time, an accumulation layer close to full occupation are hindering ion transport across the interface due to the repelling forces of the respective charge carrier.^[16]^ Hence, the interlayer leads to a smearing of the ion/vacancy distribution near the interface, which appears as an SCL broadening. By using the thickness of the SCL, d_SCL,_ as well as the resistance of the space charge layers, the calculated conductivity shows that it is about three magnitudes of order smaller (~10^−7^ S/cm) than the original bulk conductivity (1 ⋅ 10^−4^ S/cm) (cf. Figure 3D).

The interlayer can be further investigated since its thickness is fixed and volume changes are minimal for a zero‐strain material. Hence, one can calculate the change in the dielectric constant with applied potential, which follows the interlayer capacitance (cf. Figure 4B and 4C). R_IL_ is decreasing and even vanishing towards lower bias potentials, indicating a well‐conducting IL, the apparent interlayer permittivity rises simultaneously. A similar phenomenon was reported by Katzenmeier et al. but within the LICGC electrolyte. There, it is a consequence of the increasing accumulation of lithium‐ions due to much faster mass transport within the electrolyte compared to the charge transfer through the double layer^[3]^ – in this study, this seems to happen within the interlayer.

A further comparison between the two studies points to two important parameters to comprehend the findings: First would be the dielectric constant of the electrolyte and the adjacent material. While it has been shown that a lower permittivity is favored to mitigate SCL effects for the electrolyte,^[21]^ the opposite is true for the electrolyte‘s surface/interface.[^22^, ^23^] As can be seen from the impedance data, the usage of the described interlayers has a similar behavior, as the interlayer‘s permittivity sharply increases until the interlayer is conducting (cf. Figure 4C).

Secondly, current densities during characterization should increase to investigate the impact of an SCL effect closer to operational conditions. For LICGC, this is challenging due to its instability against Li metal. Thin gold layers between electrolyte and lithium can protect the LICGC from immediate degradation, and reported currents are up to ~0.13 μA/cm^2^ for bias potentials up to 1 V.^[3]^

The steady‐state current during EIS measurements in this study reaches up to ~50 μA/cm^2^ in the same potential range, increasing to ~100 μA/cm^2^ for ±2 V. Hence, the used interlayers allow to observe the SCL effect at higher currents while stabilizing the interface during the characterization.

**Figure 5:**
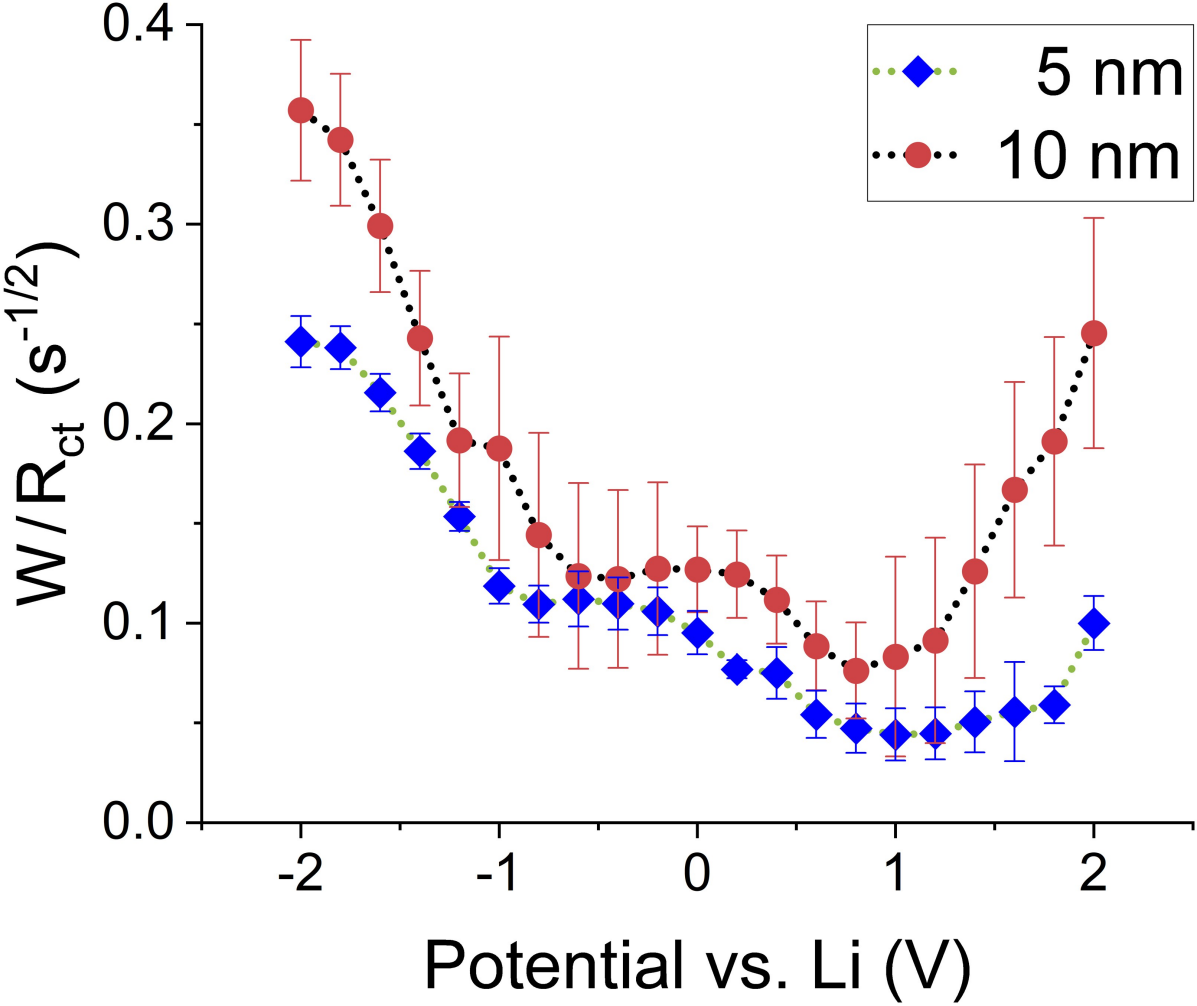
The ratio between the Warburg coefficient W and charge transfer resistance R_ct_ for both interlayer thicknesses.

To get further insight into the link between the SCL and the kinetics across the interlayer, one can use *R_ct_* and *W* to assess the apparent rate coefficient *k_app_* – the total speed at which electrochemical reactions take place at an electrode^[27]^ with respect to an “averaged” local deposition site (irrespective of the electrode surface area and surface concentration of electroactive species^[25]^). Although this evaluation was developed to evaluate the kinetics of film formation via the mentioned impedance parameters *R_ct_* and *W*,^[25]^ *k_app_* also proved useful to monitor fundamental processes at the interface in Li‐ion batteries^[26]^ and fuel cells.^[24]^ Assuming the diffusion coefficient is constant, one can investigate the ratio *W/R*_*ct*,_ which should then show the trend of *k_app_* (*k_app_* ∝ *W/R_ct_*) concerning the applied potential. Doing so, again, an asymmetry with respect to 0 V and two local minima for both interlayer thicknesses emerge (cf. Figure 5). The implication is not unexpected: The kinetics of the charge transfer across the interface is connected to the degree to which space charge layers form, as the potential for the minima in *k_app_* coincides with the SCL thickness maxima (cf. Figure 3B).With the increase in SCL thickness and, therefore, its resistance *R_SCL_*, the overall reaction at the interface (*k_app_*) is also limited. On the contrary, towards ±2 V, the SCL shrinks, which promotes the rate, and hence, *k_app_* increases.

Nevertheless, this observed SCL decrease towards +2 V and −2 V needs clarification. The SCL thickness should plateau for higher bias potentials since once the electrochemical potential of the adjacent material has been completely shielded off by charge migration, no further SCL change is expected.^[16]^ However, the SCLs shrink to a minimum (cf. Figure 3B). This new behavior may be the consequence of a complex interplay between the changing properties of the three layers at the interface: interlayer, DL and SCL. For example, towards −0.8 V, where *R_IL_* becomes negligible, the interlayer material is supposedly approaching Li_2_Ti_2_O_4_. Li^+^ must hence accumulate within the interlayer, which gives rise to the increase in the interlayer′s permittivity *ε_IL_*.^[3]^ If now the bias potential is increased to −2 V, the declining *ε_IL_* would be a consequence of draining Li^+^ and, hence, a delithiating interlayer (Li_x_Ti_2_O_4_ with x < 2). Since the dielectric properties of the interlayer material change substantially between those two potentials (cf. Figure 4C), the charge carriers within the electrolyte adjust accordingly with a broadening (−0.8 V) or a thinning (towards −2.0 V) of the SCL. For an operational ASSB full cell, the implications of these observations at negative potentials vs. Li are, of course, not applicable; nevertheless, a similar behavior is registered for positive potentials. In the next step, therefore, this interlayer should be investigated within a full cell at typical battery potentials between 3.0 V and 4.2 V.

Finally, to see how stable the as‐prepared samples are, the samples were cycled up to 1 mA/cm^2^ after the characterization phase. Although the total impedance increases with each cycle, no shortcut due to forming dendrites is detected. After disassembly, the original white solid electrolytes turned black, which is a well‐known sign of Ti^4+^ reduction to Ti^3+^.^[4]^ A crack developed at the edge of the 10 nm sample while the 5 nm sample was found completely intact, which suggests that thinner TiO_x_ layers are favored at solid‐liquid interfaces as well as between two solids, not only from a kinetic point of view but also a mechanical one.

**Figure 6:**
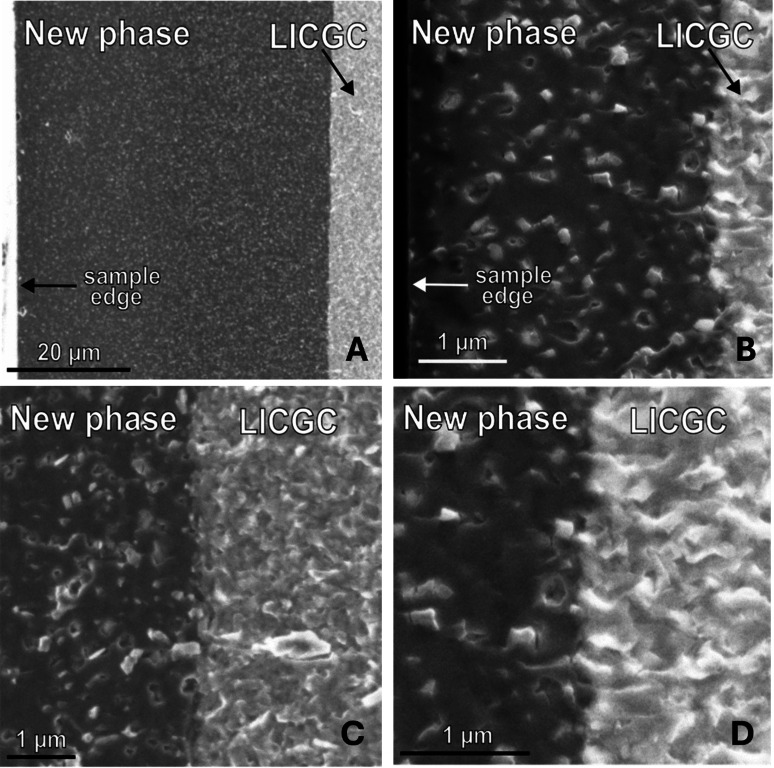
Cross‐section SEM pictures of LICGC. Surface of the 5 nm sample (A) and 10 nm sample (B). Interlayers are on the left side at the sample edge (very small visible indication for 10 nm sample only). The darker regions are the newly formed phase, which grows into the bulk electrolyte LICGC (brighter areas to the right). Close‐ups of the interfaces between the original electrolyte structure and the new phase for 5 nm (C) and for 10 nm interlayers (D).s

After this, the cross‐section of both samples was investigated *ex‐situ* by Scanning Electron Microscopy (SEM). The images reveal the formation of another phase within the solid electrolyte. Comparing overview pictures of both samples (cf. Figure 6A and 6B) shows that a thinner interlayer leads to a thicker phase growing from the surface of the electrolyte into the bulk. For the 5 nm sample, this new layer expanded to about 45 μm, while the interphase within the 10 nm sample, however, only extends ~4 μm on either side, respectively. At close‐up, by comparing the unchanged solid electrolyte ‐ where the typical grains are visible^[15]^ ‐ to the newly formed phase, it looks at first as if the latter is just the grains filled up with lithium (cf. Figure 6C and 6D).

Hartmann et al. have shown that a mixed‐conducting interphase forms if the LICGC electrolyte is unprotected from the metallic lithium. Those layers, allowing the conduction of both ions and electrons, lead to rapid interface degradation, resulting in high interfacial impedances.[^4^, ^28^] Although the oxidation of lithium within the interlayer requires both ionic and electronic conduction, here, however, even when tested for hundreds of hours, the last measurement of the sample‘s total impedance showed around 14 kΩ at 0 V. As a direct contact between lithium metal and the solid electrolyte would degrade the interface very quickly; the resulting interphase has to work in a different way as it at least slows down the degradation.

Also, the interface between the interphase and the bulk material is very homogenous and ordered, something which cannot be said about the unprotected Li/LICGC interface.^[4]^ Interestingly, Katzenmeier et al. report a non‐vanishing SCL thickness at 0 V for the LICGC with blocking[^16^, ^29^] and semi‐blocking electrodes.^[3]^ This could be an indication that similar interphases could slowly form there as well. The composition and the conditions under which this new interphase forms with the here‐used interlayers should be further examined to understand its link to the SCLs, which are also in the μm‐range. However, such a consideration is outside the scope of this study and needs further investigation.

## 3 Conclusions

This study assessed nanometer‐thin TiO_x_ layer as an interlayer for the unstable Li/LICGC interface. The initial high cell impedances and overpotential during cycling improve after a lithiation phase, as TiO_2_ turns into a stable interlayer, allowing the reversible cycling versus metallic lithium. This allows to investigate a solid electrolyte under non‐blocking conditions for a prolonged time to study the SCL effect under higher steady‐state currents. Due to that, the following observations were possible:

- SPEIS and a physical equivalent circuit were able to distinguish the bulk from the interlayer (IL), space charge layer (SCL) and double layer (DL).
- The impedance response of the interlayer could be monitored throughout the potential range. For low applied bias potentials, the interlayer′s impedance is negligible, meaning it shows good Li^+^ conduction. Towards high bias potentials, however, the interlayer resistance rises. Conversely, the dielectric constant *ε_IL_* decreases towards high bias potentials, which can be attributed to the varying lithiation degree of the interlayer.
- Space charge layers up to 5.1 μm were observed, which is a tenfold increase compared to former investigations of the same electrolyte, and simultaneously, the resistance of the SCLs was found 200x lower. Both findings point toward a less concentrated depletion SCL layer.
- It was established that the kinetics across the interface are linked to the SCL thickness. The apparent rate coefficient *k_app_* on the interface is the lowest when the SCL is the thickest.
- Additionally, *ex‐situ* SEM showed that after the characterization and cycling up to 1 mA/cm^2^, an interphase develops on either electrolyte surface, which grows into the bulk.

In conclusion, nanometer‐thin TiO_2_ layers proved to be a measure against the unstable interface between lithium metal and LICGC and, as such, enabled the prolonged characterization of symmetric all‐solid‐state lithium metal cells.

## 4 Experimental

### 4.1 Atomic Layer Deposition of TiO_2_ layers

TiO_x_ nanolayers were deposited by a flow‐type hot‐wall ASM F‐120 reactor below 3 mbar pressure and with a deposition temperature of 160 °C. The precursors were titanium isopropoxide (TTIP, Aldrich 97 %) and water as a co‐reactant. One cycle consisted of 2 s of TTIP and 1 s of H_2_O pulse, with 6 s long N_2_ purging periods in between. The exact procedure is described in a previous research work.^[30]^

The commercially available ceramic glass electrolytes (LICGC™, diameter: 19 mm, thickness: 150 μm) from Ohara Inc. (Japan) were placed onto a flat holder in the deposition chamber. Each side of the electrolyte disc was coated using either 125 or 250 cycles, which results in 5 nm or10 nm layers of TiO_x_, respectively.

A control measurement with a spectroscopic ellipsometer and a model for TiO_2_ after the deposition showed, that the cycle count translates to TiO_2_ layers of approximately 3.6 nm and 7.2 nm in thickness, respectively. Nevertheless, throughout the study, the original name of the sample remains, i. e. both will be called ′5 nm sample′ and ′10 nm sample′.

### 4.2 Preparation of Symmetric Cells

Lithium chips from MSE Supplies with a diameter of 15.6 mm and a thickness of 0.25 mm were placed onto either side of the electrolyte after scratching of the metallic lithium surface to remove any passivation layer. A LICGC disc coated with TiO_2_ was subsequently inserted between lithium chips within a PAT‐cell obtained from EL‐CELL, Germany. This cell is equipped with two polished stainless‐steel plungers, ensuring contact with both sides. One side connects to both the reference (RE) and counter electrode (CE), while the other connects to the working electrode (WE). All preparations and measurements were conducted within an argon‐filled glovebox with stringent atmospheric control (O_2_ < 0.5 ppm, H_2_O < 0.5 ppm).

### 4.3 Lithiation and Measurement Procedure

Impedance spectra were recorded between 6 MHz and 0.1 Hz with a 10 mV AC perturbation amplitude at room temperature. Within the lithiation phase, galvanostatic charge‐discharge cycles with a constant current of 0.01 mAh/cm^2^ were performed for a total of 120 hours. In between, impedance spectra at 0 V and cycling voltammograms (range: ±2 V, scan speed: 5 mV/s) assess the lithiation progression. After the cells have reached a stable state, they are characterized by Staircase Potentiostatic Electrochemical Impedance Spectroscopy (SPEIS) in the following order: 0 V→−2 V→+2 V→0 V and in 0.2 V steps, with a waiting time of 30 min at each step. The presented data stems from the −2 V to +2 V pass. Following the characterization phase, galvanostatic cycling up to 1 mA/cm^2^ serves as an extreme stability test.

## Supporting Information

Additional fitted parameters for both the 5 nm and 10 nm sample are available in the supporting information.

## Abbreviations

ASSBs: All‐solid‐state batteries
DL: Double layer
EEC: Electrochemical equivalent circuit
EIS: Electrochemical Impedance Spectroscopy
IL: Interlayer
SPEIS: Staircase Potentiostatic Electrochemical Impedance Spectroscopy
SCL: Space charge layer

## Conflict of Interests

The authors declare no conflict of interest.

5

## Supporting information

As a service to our authors and readers, this journal provides supporting information supplied by the authors. Such materials are peer reviewed and may be re‐organized for online delivery, but are not copy‐edited or typeset. Technical support issues arising from supporting information (other than missing files) should be addressed to the authors.

Supporting Information

## Data Availability

The data that support the findings of this study are available from the corresponding author upon reasonable request.
