# Supplementary material for: Characterization of the Lithium/Solid Electrolyte Interface in the Presence of Nanometer‐thin TiOx Layers for All‐Solid‐State Batteries
Source: ChemSusChem. 2024 Oct 16;17(22):e202401026. doi: 10.1002/cssc.202401026 (PMC11587694; doi:10.1002/cssc.202401026)
Supplement: Supplementary file 1 — Supporting Information [file CSSC-17-e202401026-s001.pdf]

ChemSusChem

Supporting Information

Characterization of the Lithium/Solid Electrolyte Interface in the Presence of Nanometer-thin TiO_x Layers for All-Solid-State Batteries

Rainer Götz, Ekaterina Pugacheva, Zahra Ahaliabadeh, Princess Stephanie Llanos, Tanja Kallio,* and Aliaksandr S. Bandarenka*

Supporting Information

Characterization of the Lithium/Solid Electrolyte Interface in the Presence of Nanometer-thin TiO_x Layers for All-Solid-State Batteries

Rainer Götz,[1] Ekaterina Pugacheva,[1] Zahra Ahaliabadeh,[2] Princess Stefanie Llanos,[2] Tanja Kallio,[2,] Aliaksandr S. Bandarenka[1,*]*

** Corresponding Authors:*

A.S. Bandarenka, Technical University Munich, E-mail: bandarenka@ph.tum.de

T. Kallio, Aalto University, E-mail: tanja.kallio@aalto.fi

[1] Physics of Energy Conversion and Storage, Physics Department, Technical University of Munich, James-Franck-Str. 1, 85748 Garching, Germany.

[2] Electrochemical Energy Conversion, Aalto University, P.O. Box 11000, Otakaari 1B, 00076, Aalto, Finland.

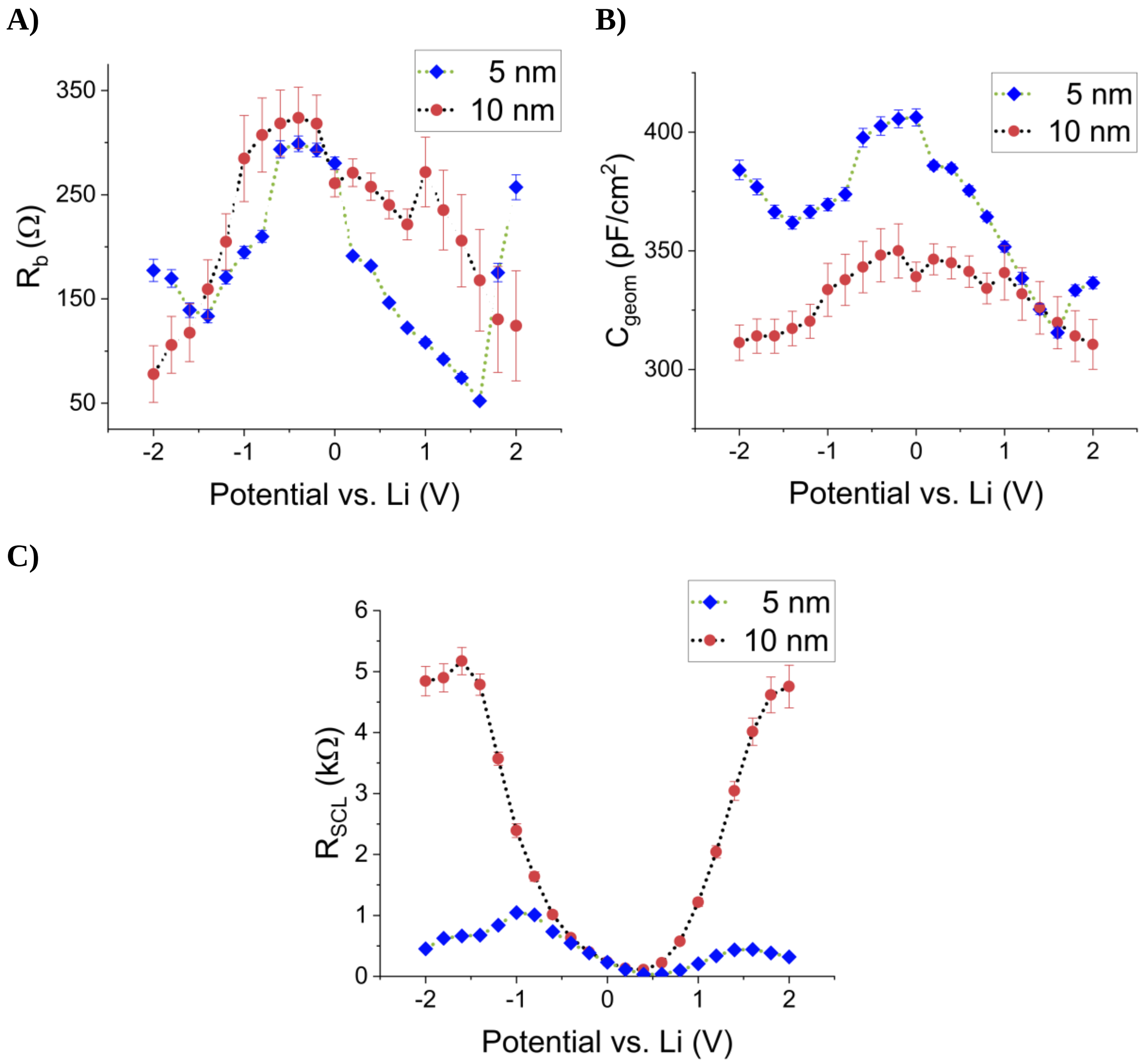

Figure S1. *Dependencies on the potential of* ***A)*** *Bulk resistance R_b,* ***B)*** *geometric capacity C_{geom} and* ***C)*** *space charge layer resistance R_{SCL} for both 5 nm and 10 nm samples*

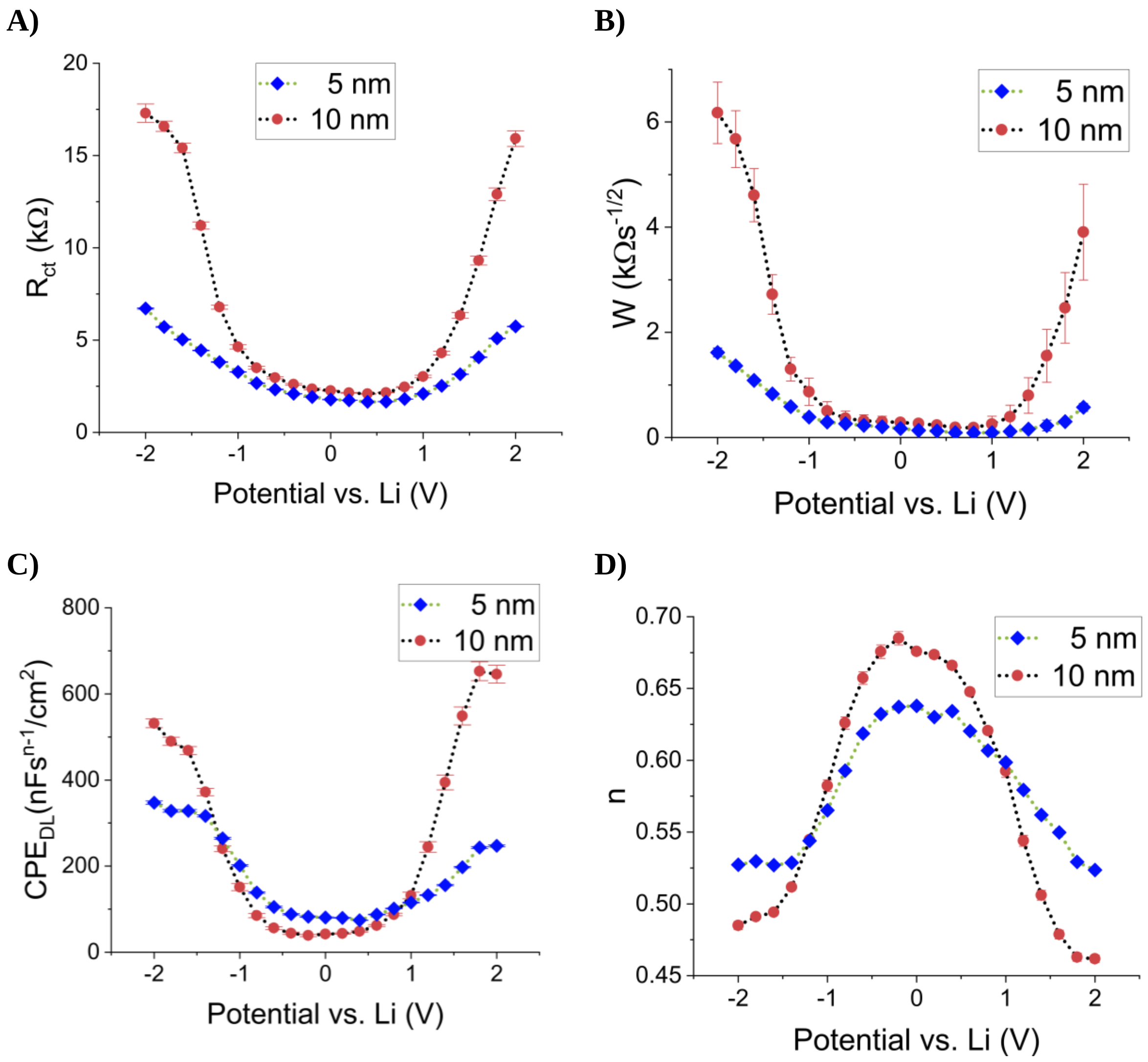

Figure S2. *Dependencies on the potential of* ***(A)*** *Charge transfer resistance* R_{ct} ***(B)*** *Warburg element for the Li diffusion across the double layer W* ***C)*** *a constant phase element assigned to the double layer* CPE_{DL} ***D)*** *and its respective exponent n for both the 5 nm and 10 nm samples*
